# Integration of child–parent screening and cascade testing for familial hypercholesterolaemia

**DOI:** 10.1177/0969141318796856

**Published:** 2018-10-14

**Authors:** David S Wald, Nicholas J Wald

**Affiliations:** Wolfson Institute of Preventive Medicine, Queen Mary University of London, London, UK

**Keywords:** Familial hypercholesterolaemia, screening, child–parent, cascade

## Abstract

**Objective:**

To integrate child–parent screening and cascade testing into a single pathway-child-parent cascade screening (CPCS), for the identification of familial hypercholesterolaemia in the population and to estimate the number of new familial hypercholesterolaemia cases identified per child screened and the associated costs.

**Methods:**

We applied the results from the published MRC Child–Parent Screening Study to 10,000 children, together with cascade testing first degree relatives of parents with a familial hypercholesterolaemia mutation identified by child–parent screening. We estimated the number of familial hypercholesterolaemia cases identified per child screened, the median cost per familial hypercholesterolaemia case identified and the median cost per child screened to identify one case using a range of cholesterol and familial hypercholesterolaemia mutation testing costs. We present a case study to illustrate the application of CPCS in practice.

**Results:**

CPCS identifies one new familial hypercholesterolaemia case per 70 children screened at a median estimated cost of £960 per new familial hypercholesterolaemia case or £4 per child screened. CPCS identifies an average of four new familial hypercholesterolaemia cases per family. In the case study, six new familial hypercholesterolaemia cases were identified, and preventive treatment started in five, with the index child expected to start when older.

**Conclusion:**

CPCS for familial hypercholesterolaemia are complementary strategies. The sustainability of cascade testing relies on identifying new unrelated index cases. This is achieved with population-wide child–parent screening. Integrated CPCS is currently better than either method of familial hypercholesterolaemia detection alone. It has the potential to identify all, or nearly all, individuals with familial hypercholesterolaemia in the population at low cost.

## Introduction

Familial hypercholesterolaemia (FH) is a common and serious cause of inherited heart attacks in the young.^1^ Early identification of affected individuals is important because cholesterol-lowering treatment is effective in preventing clinical events.^2^ Child–parent screening is sometimes compared with cascade testing for FH, as if they were alternative identification strategies, but they are not.^3^ Child–parent screening is a method of population screening that identifies unrelated FH families.^4^ Cascade testing is a clinical activity that identifies related FH individuals within families.^5^

Cascade testing from an index case identifies one new FH case per two relatives tested, but identifies only a small proportion of all cases in the population, because it is limited by the number of known unrelated index cases.^6^ Child–parent screening is not limited in this way but needs to screen about 250 children to identify one new unrelated FH case.^4^ These become index cases for cascade testing. Such a source of known unrelated FH cases is needed for cascade testing to be sustained. Child–parent screening and cascade testing thus need to be considered together, with the latter dependent on the former for about three decades (one reproductive generation), when most unrelated families with FH in the population will have been identified by screening.^6^

We here describe and evaluate the integrated strategy in which child-parent screening and cascade testing are regarded as two separate phases of a single pathway, child-parent cascade screening (CPCS). We estimate the number of new cases that can be identified per child screened, together with the associated costs. We illustrate the CPCS strategy with a real case study, where cascade testing followed naturally from child–parent screening in the MRC Child–Parent Screening Study and propose that this becomes the model adopted in practice.

## Methods

For the child–parent screening phase, we used the protocol based on the results of the MRC Child–Parent Screening Study.^4^ Total cholesterol is measured in children at the time of routine immunisation at about 12 months of age, followed by FH mutation testing in children with a cholesterol level at or above 1.35 multiples of the median (MoM) – the top 5% of values. Children without an FH mutation and a total cholesterol measurement at or above 1.5 MoM (the top 1% of values) have a repeat total cholesterol measured about three months later, combined with a subsequent routine immunisation, e.g. pneumococous. For each positive child, one FH-positive parent is identified either with the same FH mutation as their child or, in the absence of a mutation, with the higher of the two parental cholesterol values, a protocol that correctly identifies the FH parent in five of six parent pairs,^4^ but would miss the detection of one parent in the rare situation in which both parents had FH. For the cascade testing phase, we assumed families had on average two children (average according to the Office for National Statistics for the past two decades),^7^ and testing was limited to families with an identified FH mutation and to first degree relatives (i.e. sibling of the affected child, sibling of the affected parent, and parents of the affected parent). In this way, a further four relatives are tested, half having the FH mutation, who are regarded as FH positive. We assumed that all relatives were available and agreed to testing.

## Results

**Figure 1.**
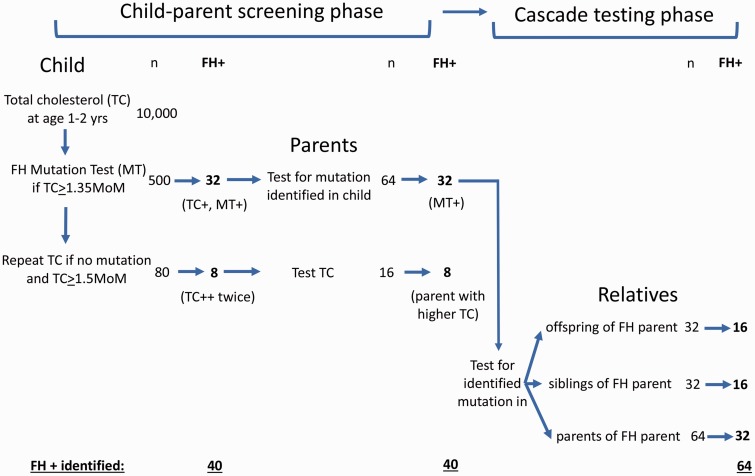
Integrated child–parent screening and cascade testing showing the number of tests (n) and FH-positive cases (FH+) identified based on screening 10,000 children. FH: familial hypercholesterolaemia.

Figure 1 shows the CPCS pathway based on screening 10,000 children. In the child–parent screening phase, 40 FH-positive children are identified (32 with a total cholesterol ≥1.35 MoM and an FH mutation, plus eight with two total cholesterol levels ≥1.5 MoM, three months apart), together with 40 FH-positive parents (32 with the same FH mutation as the affected child and eight with the higher of the two parental cholesterol measurements), resulting in 80 FH-positive cases. A further 64 FH-positive individuals are identified in the cascade testing phase by testing within the 32 separate families with an identified FH mutation, and classifying a relative as positive for FH based only on the presence of the FH mutation. A total of 144 FH-positive individuals are thus identified per 10,000 children screened, or about one new FH-positive individual per 70 children screened.

Table 1 shows the cost per case identified and the cost per child screened to identify one FH case. For screening children, we used unit costs for cholesterol measurement (point-of-care testing) ranging from £1 to £5 and for FH mutation testing ranging from £50 to £400. FH mutation testing in parents and other relatives of an index child required testing only for the FH mutation found in the child, at £50 per test and cholesterol at £5 per test. The median cost per case identified was about £960 and the median cost per child screened to identify one FH case was about £4 (range about £300 to £1780 and £1 to £7, respectively).

**Table 1.** Integrated child–parent screening and cascade testing (CPCS) – Cost per case identified and cost per child screened.

	FH mutation test				
	£50	£100	£200	£300	£400
Cholesterol test	Cost per case identified (cost per child screened)^a^				
£1	304 (1.2)	477 (1.9)	825 (3.3)	1172 (4.7)	1519 (6.1)
£2	373 (1.5)	547 (2.2)	894 (3.6)	1241 (5)	1588 (6.4)
£3	443 (1.8)	616 (2.5)	963 (3.9)	1311 (5.2)	1663 (6.7)
£4	512 (2)	686 (2.7)	1040 (4.2)	1380 (5.5)	1715 (6.9)
£5	582 (2.6)	750 (3)	1102 (4.4)	1450 ( 8)	1784 (7.1)

a Cost per case identified/250.

## Discussion

The CPCS strategy can identify about one new FH individual for every 70 children screened and provides a flow of unrelated index cases from whom cascade testing can be sustained, yielding an estimated four new FH cases per family (three FH-positive relatives per affected child).

The method begins by screening the population at an age (between one and two years) when a total cholesterol measurement is most accurate in identifying individuals with FH.^8^ In newborns and adults, the overlap in the distributions of total cholesterol in affected and unaffected individuals is known to be greater, which makes screening at these ages less effective.^8^ Age 12 months is when children are already attending general practice for immunisation, e.g. *Haemophilus influenzae*, providing a convenient screening turnstile with the potential for universal coverage of the population. Also, by screening children, most FH-positive adults subsequently identified by cascade testing are relatively young (average age of parent/sibling of parent is about 30 years),^4^ providing an opportunity for preventive treatment before the onset of ischaemic heart disease events.

The classification of an FH-positive person during the child–parent screening phase differs from that in cascade testing phase of CPCS, the former relying on a combination of high cholesterol and an FH mutation (or two very high cholesterol levels several months apart) and the latter relying only on finding an FH mutation. This is because an FH mutation alone is not sufficient to define FH in screening the population for new cases (about one-third of children with an FH mutation do not have high cholesterol) and because in adults a cholesterol measurement is a poor way of identifying FH due to the greater overlap in the distributions of cholesterol measurements in affected and unaffected persons. While relying on the presence of an FH mutation alone in the cascade testing phase will sacrifice some detection, it will identify most cases, and it makes the tracking strategy clear (a relative either has or does not have the FH mutation). It may also be sensible to offer cholesterol testing to the siblings of FH-positive children without a mutation. While this has not been tested in practice, and is, therefore, not part of the CPCS pathway, clinicians may think it would be sensible to use cut-offs of ≥1.5 MoM on two separate occasions about three months apart.

**Figure 2.**
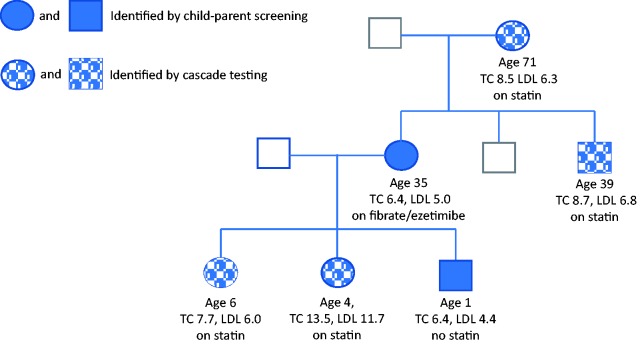
Case study of integrated child–parent screening and cascade testing. Ages to nearest year at time of testing FH positive. Total cholesterol (TC) and LDL-cholesterol (LDL) in mmol/L. All cases had the same FH mutation in LDLR gene (c.2093_2094dup). Medication started following identification is indicated.

Figure 2 shows the family tree of a child, aged 13 months, who participated in the Child–Parent Screening Study^4^ in 2014 and was identified as a positive FH case. The total cholesterol level was 6.4 mmol/L (1.62 MoM) and an FH mutation was identified in the LDLR gene (c.2093_2094 duplication). His mother, aged 35, had a total cholesterol of 6.4 mmol/L and the same mutation. Cascade testing from these individuals led to the identification of a further four FH-positive relatives, all with the same FH mutation. All affected relatives started cholesterol-lowering treatment (statins in four and fenofibrate/ezetimibe in one), including two siblings (aged four and six years) with total cholesterol levels of 13.5 mmol/L and 7.7 mmol/L, respectively. The level of cholesterol in the child aged four was so high that sequencing of all known FH genes was undertaken, even after testing positive for the specific LDLR mutation by targeted testing, in case she had more than one mutation. Only the LDLR mutation was present, supporting the observation that the same mutation can lead to quite different cholesterol levels in different people.^4^ Figure 2 illustrates the value of the CPCS strategy; five new FH-positive individuals were identified from one child detected by screening.

Cascade testing from an FH-positive child is likely to reach more relatives than cascade testing from an adult, because the relatives are younger, more accessible, and perhaps more motivated, given that the index case is a child. In England, the number of new cases identified per known case, based on cascade testing from adults, is less than one^9–11^ compared with an expected average of about three in the integrated pathway described here.

The costs of child–parent screening are low when performed at the time of childhood immunisation, because it uses an existing primary care infrastructure. The children and parents are already visiting their doctor and the screening and immunisation procedures are performed simultaneously, thereby avoiding extra clinic visits. Two generations are screened together, and this triggers cascade testing. The median cost per FH-positive case identified from the CPCS strategy is about £960, the precise cost depending on the charge for cholesterol and FH mutation testing (Table 1), which should be affordable in most healthcare systems. In 2008, The National Institute for Health and Care Excellence (NICE) judged cascade testing to be cost-effective. Combined with child–parent screening, it would be both cost-effective and sustainable.^12^ NICE assumed testing costs for cholesterol of £1.60 per sample and for FH mutation testing (sequencing the *LDLR, APOB* and *PCSK9* genes) of £400 per sample.^12^ Applying these charges to integrated CPCS means the cost per case identified would be about £1560 and the cost per child screened about £6 – less than the cost of administering one influenza vaccination (£9.80).^13^ The costs of CPCS will decline as the cost of FH mutation testing declines. For example, in California, sequencing of *LDLR, APOB* and *PCSK9* is currently available for $100 (£74).

Cholesterol-lowering treatment will, in most cases, rely on statin therapy, which is available as a generic and therefore inexpensive medicine. Annual cholesterol monitoring once treatment is started, together with prescription of drug therapy are activities that, for most patients, can be managed in primary care. In FH screening, it would be useful to establish a register to remind general practitioners and parents to consider starting treatment when their child reaches about age 10. This could either be organised on a national basis (e.g. the UK) or in separate Health Maintenance Organisations (e.g. the US).

It is through screening children that adults are most effectively identified, and the sustainability of cascade testing depends on identifying new unrelated affected families, which child–parent screening achieves. We therefore suggest that consideration be given to revising current clinical guidelines,^14–16^ with a view to adopting the integrated CPCS strategy.
